# Effect of Atypical Antipsychotics on Fetal Growth: Is the Placenta Involved?

**DOI:** 10.1155/2012/315203

**Published:** 2012-07-11

**Authors:** Sandeep Raha, Valerie H. Taylor, Alison C. Holloway

**Affiliations:** ^1^Department of Pediatrics, McMaster University, 1200 Main Street West, Room 3N11, Hamilton, ON, Canada L8N 3Z5; ^2^Department of Psychiatry, Women's College Research Institute, University of Toronto, Toronto, ON, Canada M5S 1B1; ^3^Department of Obstetrics and Gynecology, McMaster University, 1200 Main Street West, Room 3N52, Hamilton, ON, Canada L8N 3Z5

## Abstract

There is currently considerable uncertainty regarding prescribing practices for pregnant women with severe and persistent psychiatric disorders. The physician and the mother have to balance the risks of untreated psychiatric illness against the potential fetal toxicity associated with pharmacological exposure. This is especially true for women taking atypical antipsychotics. Although these drugs have limited evidence for teratological risk, there are reports of altered fetal growth, both increased and decreased, with maternal atypical antipsychotic use. These effects may be mediated through changes in the maternal metabolism which in turn impacts placental function. However, the presence of receptors targeted by atypical antipsychotics in cell lineages present in the placenta suggests that these drugs can also have direct effects on placental function and development. The signaling pathways involved in linking the effects of atypical antipsychotics to placental dysfunction, ultimately resulting in altered fetal growth, remain elusive. This paper focuses on some possible pathways which may link atypical antipsychotics to placental dysfunction.

## 1. Introduction

Women of childbearing age appear to be particularly susceptible to the exacerbation of existing mental illness and the development of new mental illness [1–5]. Indeed, it has been estimated that over 500,000 pregnancies annually are complicated by psychiatric illness that either precedes pregnancy or arises during pregnancy [6]. Untreated psychiatric disease during pregnancy is associated with increased risks for the mother (including self-harm/suicide, self-neglect, and reduced compliance with prenatal and postnatal care) and risks for the child (including impaired fetal development, infanticide, and impaired mother-child bonding) (reviewed in [7, 8]). Historically, antipsychotics have been extensively and effectively used for the treatment of schizophrenia and bipolar disorder, and more recently they are becoming part of the treatment of depression [9–12]. Conventional antipsychotics (also known as typical or first generation antipsychotics) which were more commonly used to treat these conditions caused a significant decrease in fertility [13]. However, the newer atypical antipsychotics do not have this side-effect. As a consequence, the number of women taking antipsychotics, who are becoming pregnant, is on the rise. Indeed, appointments at a Motherisk Program Clinic related to the use of antipsychotic medications increased 170% between 1989 and 2001; a rise which was for the most part attributable to an increased use of second-generation or atypical antipsychotics [14]. The vast majority of women who use antipsychotics during pregnancy do so because of ongoing illness. In fact, only in cases of where the first schizophrenic episodes [15] are reported in pregnancy or there is a risk of puerperal psychosis [16] would the exposure of antipsychotic exposure be restricted to the pregnancy period.

Functionally, typical antipsychotics exhibit high affinities for D_2_ receptors [15, 17] in the mesolimbic, mesocortical [16], and nigrostriatal dopamine pathways. This, rather nonspecific targeting of dopaminergic pathways, can result in a range of undesirable motor disorders [18]. Atypical antipsychotics, however, are more selective for the D_2_ receptors in mesolimbic pathway as compared to those in the mesocortical and nigrostriatal pathways. In addition, they also target the serotonin receptor subtype 2A (5HT_2A_) [19] which may help to reduce the negative side-effects associated with typical antipsychotics [19, 20]. It is important to note that the proper correlation between the ratio of the clinical dosage and D_2_ receptor affinity necessary to treat the symptoms of conditions such as schizophrenia has not been completely established [21]. 

It is well understood that the suitability of antipsychotic medications during pregnancy is a balance between the risks of adverse obstetrical and neonatal outcomes and the risks associated with untreated or inadequately treated psychiatric illness. The most common atypical antipsychotics administered during pregnancy are Olanzapine, Clozapine, Risperidone, Quetiapine, and Aripiprazole [22]. Complicating the matter further is that almost 40–57% of women taking atypical antipsychotics during are prescribed a combination of these drugs (polytherapy) [23–25]. In general, current practice guidelines discourage changing medications during pregnancy as this may leave the patient on nontherapeutic doses during a period of time; a situation which is not in the best interests of the mother. Therefore, the usual standard of care dictates that dosages be increased or polytherapy be implemented. 

Although general clinical practice guidelines have been established (ACOG, 2008) [9], there remains significant uncertainty regarding effects of these drugs on the fetus. To date, the teratogenic effects of antipsychotic exposure have received significant attention; however, the effects of these medications on long-term health outcomes of the offspring have not been well studied. In adults, one of the major side-effects of antipsychotic use is the dysregulation of body weight homeostasis (reviewed in [26, 27]). Similarly, maternal use of antipsychotics has been reported to result in aberrant fetal growth. Indeed maternal antipsychotic use has been reported to result in an increased incidence of both low and high birth weight relative to the general population [23, 24, 28, 29]. Since being either too small or too large at birth is a risk factor for the development of metabolic syndrome in postnatal life [30–32], children exposed to antipsychotic medications *in utero* may be at increased risk of developing obesity in postnatal life. However, there are no human studies which have tested this hypothesis and the mechanism(s) by which atypical antipsychotics may affect fetal growth have yet to be elucidated. It is very difficult to decipher, based on the available literature, why atypical antipsychotics can cause both small for gestational age (SGA) and large gestational age (LGA) fetuses. For example, McKenna et al. [23] report an increased risk of SGA in women taking the atypical antipsychotics Olanzapine, Risperidone, Quetiapine, and Clozapine. In contrast, Newham et al. [24] report an increased risk of LGA for women taking the atypical antipsychotics Amisulpride, Clozapine, Olanzapine, Quetiapine, and Risperidone. Since the sample sizes in these studies are small for women taking each of the individual drugs, the risks associated with each drug cannot be accurately reported. Furthermore, the paucity of data evaluating the action of antipsychotics in pregnancy using animal models makes the discussion of mechanisms for these drugs difficult.

## 2. Maternal Antipsychotic Use and Altered Birth Weight: Mechanisms

### 2.1. Atypical Antipsychotics and Maternal Metabolic Derangement

It is well accepted that atypical antipsychotic use can alter body weight homeostasis in humans and nonpregnant rodent models, resulting in significant drug-induced weight gain and visceral fat accumulation [26, 27, 33], an effect which is more pronounced in females [34]. The weight gain varies among the atypical antipsychotics, ranging from 2 to in excess of 25 kg [27, 35], and can affect up to 60% of patients after 3–12 months of use [34]. Furthermore, there is now considerable evidence from animal experiments and clinical studies that the use of atypical antipsychotics is a major risk factor for impaired glucose homeostasis and type 2 diabetes in the nonpregnant population [26, 27, 36–40]. However, the effects of atypical antipsychotics on gestational weight gain, postpartum weight retention, and gestational diabetes have not been systematically addressed; although, one study has reported that the use of antipsychotics during pregnancy significantly (OR 1.78, 95% CI 1.04–3.01) increases the risk of developing gestational diabetes [41]. In addition to metabolic consequences of antipsychotic medications, there are a number of other risk factors which may explain the increased incidence of diabetes in mentally ill patients including hereditary factors, poverty and poor access to good nutrition, physical inactivity, and antipsychotic medication use (reviewed in Holt et al., 2004 [42]). These changes in glucose homeostasis may explain, in part, the increased incidence of large for gestational age (LGA) babies born to women taking atypical antipsychotics during pregnancy as LGA is regarded as the most common fetal complication when women have GDM or preexisting diabetes [43–46].

The mechanisms by which atypical antipsychotics can cause maternal obesity and dysglycemia are varied, and the relative contribution of each pathway to an aberrant metabolic state is not completely understood. Atypical antipsychotic-induced weight gain and fat accumulation may be the result of altered food intake (i.e., hyperphagia and decreased satiety) [47–50], direct effects of the drug on adipocytes to alter lipogenesis and lipolysis in favour of lipid accumulation [51–53], or effects on peripheral tissues to induce insulin resistance [51, 54, 55]. This insulin resistance in combination with increased gluconeogenesis [51, 54, 56] and impaired insulin secretion from the pancreatic beta cell [55, 57, 58] may in turn be responsible for the observed increase in type 2 diabetes with the use of atypical antipsychotics. However, there is a great deal of heterogeneity amongst the responses evoked by different antipsychotics. Indeed, while some evoke insulin release, others such as Risperidone, Ziprasidone, and Quetiapine do not [59]. The paucity of data using individual antipsychotics makes it difficult to conclude how these drugs induce such heterogeneous responses. Regardless of the mechanism, aberrant maternal glucose control and maternal obesity can have significant implications for the long-term health of both the mother and her child. 

**Figure 1:**
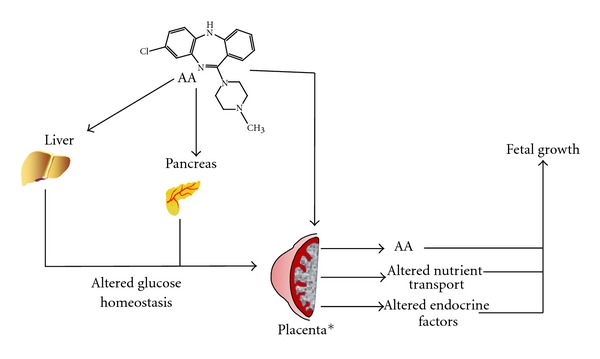
Atypical antipsychotics may impact fetal growth, by altering placental function. Atypical antipsychotics (AA) such as Clozapine are known to effect liver and pancreas function. Such effects can result in altered systemic glucose levels. During pregnancy, this can result in gestational diabetes or contribute to increased nutrient transport across the placenta. In addition, AA can be directly transported across the maternal-fetal interface and potentially impact fetal metabolic balance. However, AAs in the maternal system could impact placental development or function and alter the release of endocrine factors which would impact on fetal growth and development. *Adapted from [72].

There is compelling evidence that obesity in women, whether as a result of prepregnancy obesity, excessive gestational weight gain, and/or postpartum weight retention, is associated with an increased risk for many pregnancy-related health complications such as gestational diabetes and hypertensive disorders of pregnancy [60, 61]. In addition, maternal obesity also considerably increases the risk of fetal complications such as spontaneous abortion, stillbirth, congenital anomalies, neonatal death, and altered fetal growth. Indeed, in humans, obesity during pregnancy is associated with a significantly increased risk of macrosomia (commonly defined as birth weight ≥4500 g (8 lb 13 oz to 9 lb 15 oz)) as well as an increased risk of delivering a low birth weight baby [43, 60, 62–66]. Similarly, animal studies have also shown that maternal overweight and obesity results in altered fetal growth with reports of both increased and decreased birth weight [30, 67–69]. Therefore, based on the well-documented relationships between maternal obesity/diabetes and altered fetal growth, it is plausible that the altered fetal growth in women taking atypical antipsychotics may be a reflection of antipsychotic-induced changes in maternal metabolic or nutritional status [70]. In addition to the impact of maternal metabolic status on the pregnancy, atypical antipsychotics may also be transferred across the placenta and directly impact fetal growth. While there is limited evidence quantifying the extent to which these drugs are transferred across the placenta, the work of Schenker et al. [71] suggests that somewhere in the range of 5–14% of a labeled olanzapine can be transferred from maternal to fetal system in 4 h. However, the effects of atypical antipsychotics on fetal growth may be also mediated via altered placental development and/or function (Figure 1).

### 2.2. The Placenta as a Target of Atypical Antipsychotics

Prospective and retrospective analyses of maternal antipsychotic use and fetal outcomes have provided evidence that the use of atypical antipsychotics during pregnancy can result in dysregulated fetal growth. The results from these studies are conflicting; with some studies reporting that maternal use of atypical antipsychotics results in an increased incidence of high birth weight babies relative to the normal population and some studies reporting an increased incidence of low birth weight babies [23, 24, 28, 29]. Such observations suggest the actions of atypical antipsychotics on fetal growth may be described as a “U” shaped growth curve. Understanding the mechanisms driving such complex interrelationships may prove to be difficult, but the most likely place to initiate this search may be placental development and function.

Mechanistically, it is known that atypical antipsychotics can act via dopamine D_2_ or serotonin 5-HT_2A_ receptors [73]. In addition to their well documented, but rather heterogeneous effects on various regions of the brain, the atypical antipsychotics also affect a number of the other tissues. Recent work from Viau and colleagues reports the identification of the 5-HT_2A_ serotonin receptor in human trophoblasts [74], a lineage of cells which are of central importance to placental development and function. However, the role of this receptor in placental development and function remains unclear. In other cell types, signaling via the 5-HT_2A_ receptor has been shown to affect cellular differentiation, proliferation, and migration, all of which are central to the function of placental trophoblast cells in the establishment of proper placentation [75, 76]. Such a relationship becomes more relevant because serotonin is synthesized *de novo* in human trophoblast cells where it likely serves an important endocrine, paracrine, and autocrine role in regulating placental function [77]. Indeed, treatment of cultured trophoblasts, BeWo and JEG-3 cells, with serotonin results in increased aromatase activity; an effect which may result in altered estrogen biosynthesis [77]. Control of estrogen biosynthesis is important not only for successful implantation of the blastocyst but also for the regulation of leptin expression (review in [78]). Such regulation may be important in modulating fetal metabolism [79] and organ development [80]. Furthermore, the levels of leptin in both maternal and fetal plasma have been associated with the regulation of fetal growth [81, 82]. Taken together, this evidence raises the possibility that atypical antipsychotics may affect fetal growth through the regulation of estrogen biosynthesis and leptin expression.

Dopamine receptors, another target of atypical antipsychotics, are also known to be present in the human placenta [83], in trophoblast cells [84]. Two subtypes, D_1_ and D_2_ receptors, have been localized to the spongiotrophoblasts and giant cells of the junctional zone of rat placenta at gestational days 12–16 (term 21–23 days). These cell lineages play important roles in the establishment, development, and maintenance of pregnancy in rodents [85, 86]. Like the serotonin receptor, D_2_ receptors have also been linked to important hormone regulatory processes such as the inhibition of basal and hormone stimulated release of human placental lactogen (hPL) from trophoblast cells [87]. Furthermore, the observation that the pattern of D_2_ receptor expression varies during the course of a normal pregnancy argues for an important role for dopamine signaling during placental/fetal development [87, 88]. While the function of these receptors have not been directly related to altered fetal growth, their presence in the placenta suggests that drugs which target these receptors may have potential regulatory consequences for placental function and fetal development. While compounds such as Clozapine also have a relatively high affinity for the dopamine D_4_ receptor subtype, [89] which has been detected in the placenta [90], the functional role(s) of these receptors in placenta have not been explored.

## 3. Atypical Antipsychotics and Fetal Growth: The Role of the Placenta

There is currently a paucity of data supporting the direct action of atypical antipsychotics on placental function. However, it is biologically plausible that this class of drugs might impact placental function given the presence of the putative receptors for these drugs on trophoblast cells as discussed above. Moreover, activation of dopamine receptors by other drugs, namely, bromocriptine, has been shown to alter the expression of Pit-1; a pituitary-specific transcription factor which is synthesized in the rat placenta and is involved in the regulation of rat placental lactogen (rPL) gene expression, a hormone known to impact fetal development [91]. Alternatively, atypical antipsychotics may affect placental development and/or function via alterations in oxidative stress in this tissue.

## 4. Oxidative Stress: A Potential Mode of Action for Atypical Antipsychotics

Oxidative stress is a term used to describe the imbalance between the production of reactive oxygen species and the ability of the cell to limit their damage. There is some evidence that Olanzapine and Quetiapine may act as antioxidants in cultured peripheral neurons by decreasing oxidative stress associated with an increased accumulation of *β*-amyloid protein [92, 93] and can even work to reverse the effects of compounds known to increase oxidative stress [94]. However, the majority of evidence suggests that atypical antipsychotics are associated with increased oxidative stress [95, 96]. These, apparently, divergent effects of atypical antipsychotics may once again reflect the paucity of data in this area of research. Systematic investigation of these drugs on both neuronal and uterine systems will be important understanding the effects of these drugs on cellular stress response pathways.

**Figure 2:**
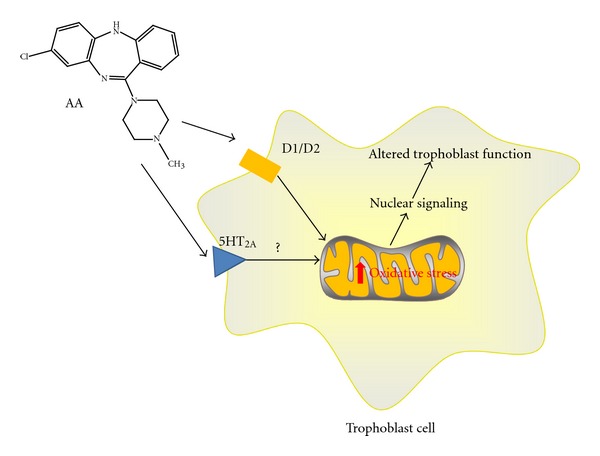
Trophoblast cells contain serotonergic and dopaminergic receptors. Trophoblast cells, which are of central importance to placental development and function, contain 5-HT_2A_ and D_1_, D_2_, and D_4_ receptors. All of these are pharmacological targets of atypical antipsychotics (AA). While the role of the D_4_ receptor in mediating the effects of AA is not currently well understood, the D_1_ and D_2_ have been associated with mitochondrial dysfunction. Mitochondrial dysfunction has been linked to increased trophoblast oxidative stress and altered fetal growth. This may be mediated in part through changes in the released levels of endocrine factors.

Clozapine, Olanzapine, and Aripiprazole have been found to differentially evoke oxidative stress in different regions of the brain [97]. For example, chronic treatment (28 days) of rats with Clozapine increased oxidative damage in the hippocampus. Olanzapine and Aripiprazole actually decreased oxidative damage in the prefrontal cortex, despite the observation that the Aripiprazole increased mitochondrial superoxide formation, a radical species known to cause both mitochondrial and cytosolic oxidative damage [98, 99], in the same tissue. Atypical antipsychotics have also been linked to increased superoxide formation and apoptosis in naïve neutrophils [100]. Furthermore, Clozapine treatment of isolated lymphoblasts increased oxidative damage of a limited group of proteins. Many of these proteins were associated with cellular energy metabolism [95]. The increased damage of proteins associated with cellular metabolism suggests that the oxidative stress may arise from the mitochondria; known to be a primary producer of cellular superoxide [101]. Production of free radicals from mitochondrial sources tends to preferentially damage mitochondrial proteins [101], the majority of which are associated with energy metabolism. Evidence that mitochondria are a target of atypical antipsychotics arises from the observation that the cerebral cortex and hippocampus of rats exposed to Clozapine exhibited altered mRNA expression for 14 mitochondrial proteins. Six of these proteins comprise various subunits of the electron transport chain, including complex I as well as complex V (ATP synthase) [102]. This is particularly pertinent to oxidative stress since inhibition of the mitochondrial respiratory chain inhibition is known to be associated with increased oxidative stress [101, 103]. While typical and atypical antipsychotics have both been shown to inhibit mitochondrial complex I activity [104, 105], the inhibitory effects of atypical antipsychotics generally require higher concentrations [104]. There are only minimal inhibitory effects of antipsychotics reported on complex II of the mitochondria [104, 106, 107]. Complex III and IV functions do not appear to be affected [108]. In addition, activation of the 5-HT_2A_ receptor has also been associated with mitochondrial biogenesis [109]. Taken together, it is feasible to propose that some of the actions of the atypical antipsychotics may occur via mitochondrially generated oxidative stress in the placenta especially since the placenta is characterized by particularly high levels of mitochondria [110]. Increased oxidative stress along with poor mitochondrial function in the placenta has been linked to growth restriction and premature fetal demise [111]. Mechanistically, oxidative stress may impact the ability of trophoblasts to transport nutrients between maternal and fetal circulation or carry out their endocrine functions [112]. However, the exact linkage between placental oxidative stress and altered fetal growth and development remains undiscovered (Figure 2). 

Oxidative stress and endoplasmic reticulum (ER) stress have been linked and may jointly influence a number of cellular processes [19, 113, 114]. However, there has been very little work done to determine whether antipsychotics impact cellular ER stress. Research by Kurosawa et al. suggests that the atypical antipsychotic Olanzapine alleviates chemically induced ER stress in cultured neurons [18]. Whether such mechanisms are relevant in *in vivo* models has not yet been explored. In addition to their effects on neuronal function, these drugs also impact reproductive functions such as fertility [115] and may also effect placental function. The mechanisms by which these drugs directly, as well as indirectly (via modulation of metabolic homeostasis), influence fetal programming need to be more thoroughly investigated.

## 5. Conclusions

There is currently considerable uncertainty regarding prescribing practices for pregnant women with severe and persistent psychiatric disorders. The physician and the mother have to balance the risks of untreated psychiatric illness, during pregnancy, against the potential fetal toxicity of atypical antipsychotic medications. Atypical antipsychotics are suspected to be teratogenic, although there are no randomized controlled studies for obvious ethical reasons [116]. The adverse metabolic side-effects of these medications compound the risks associated with teratogenicity. Given that the number of women exposed to atypical antipsychotic drugs during pregnancy is increasing, it is important to more clearly delineate the risks associated with the administration of these drugs. There is limited information regarding their impact of these compounds on placental development and/or function. The use of appropriate animal models may be crucial in understanding the effects of these drugs on fetal growth and development which have profound consequences for the long-term health of the offspring.
